# Supplementary material for: COVID-19 pathophysiology may be driven by an imbalance in the renin-angiotensin-aldosterone system
Source: Nat Commun. 2021 Apr 23;12:2417. doi: 10.1038/s41467-021-22713-z (PMC8065208; doi:10.1038/s41467-021-22713-z)
Supplement: Supplementary file 1 — Supplementary Information [file 41467_2021_22713_MOESM1_ESM.pdf]

COVID-19 pathophysiology may be driven by an imbalance in the Renin-Angiotensin-Aldosterone System

SUPPLEMENTARY

Susanne Rysz,[1,2] Jonathan Al-Saadi,[3] Anna Sjöström,[4,5] Maria Farm,[4,5] Francesca Campoccia Jalde,[2,4] Michael Plattén,[3,6] Helen Eriksson,[7] Margareta Klein,[4,8] Roberto Vargas-Paris,[4,9] Sven Nyrén,[4,9] Goran Abdula,[4,10] Russell Ouellette,[3,6] Tobias Granberg,[3,6] Malin Jonsson Fagerlund,[2,11] Johan Lundberg,[3,6]*

1. Department of Medicine Solna, Karolinska Institutet, Stockholm, Sweden.
2. Function Perioperative Medicine and Intensive Care, Karolinska University Hospital, Stockholm, Sweden.
3. Department of Clinical Neuroscience, Karolinska Institutet, Stockholm, Sweden.
4. Department of Molecular Medicine and Surgery, Karolinska Institutet, Stockholm, Sweden.
5. Department of Clinical Chemistry, Karolinska University Hospital, Stockholm, Sweden.
6. Department of Neuroradiology, Karolinska University Hospital, Stockholm, Sweden.
7. Department of Sociology, Stockholm University Demography Unit, Stockholm University, Stockholm, Sweden.
8. Department of Radiology Huddinge, Karolinska University Hospital, Sweden.
9. Department of Radiology Solna, Karolinska University Hospital, Stockholm, Sweden.
10. Department of Clinical Physiology, Karolinska University Hospital, Stockholm, Sweden.
11. Department of Physiology and Pharmacology, Karolinska Institutet, Stockholm, Sweden.

*Corresponding author:

Johan Lundberg
Department of Neuroradiology, Karolinska University Hospital, Solna, B5:19
SE-171 76 Stockholm
phone (cell): +46 73 625 8594
j.lundberg@ki.se

Supplementary Figure 1. Physiological measurements for all swines.

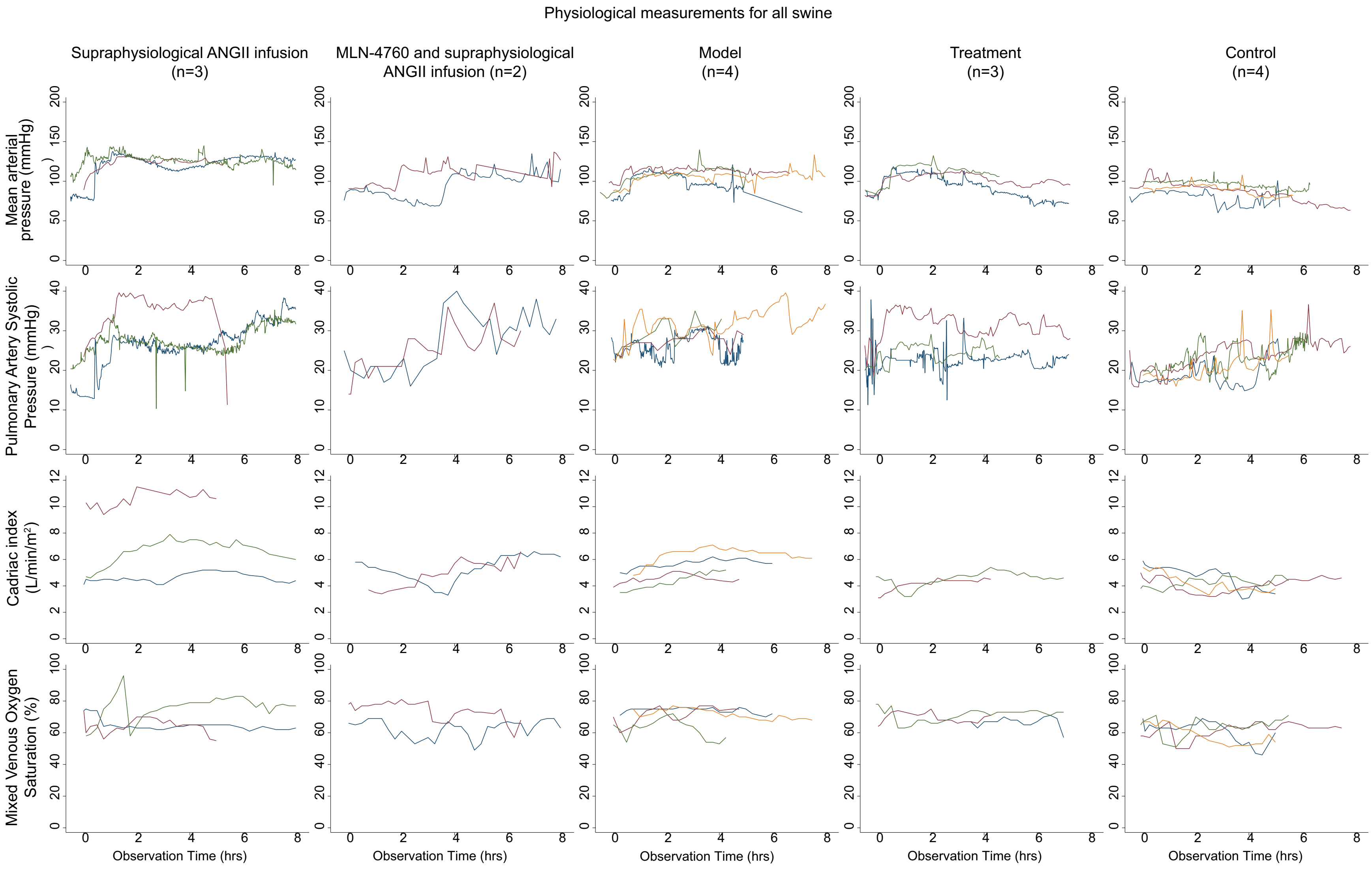

Supplementary Figure 1.

The first column represents the swine infused with supraphysiological ANGII, the second column is MLN-4760 and supraphysiological ANGII infusion, the third column is MLN-4760 and low dose ANGII, the fourth column is MLN-4760, low dose ANGII and treatment with losartan and low molecular weight heparin and finally, the fifth column are control animals. Each individual is color-coded with the same color in the groups.

First row: Invasive mean arterial pressure measured in the aorta. **Second row:** Pulmonary systolic arterial pressure measured by Swan-Ganz catheter. **Third row:** Cardiac index measured by Swan-Ganz catheter. **Fourth row:** Mixed venous oxygen saturation measured by Swan-Ganz catheter. Source data are provided as a source data file.

Supplementary Figure 2. Blood gas analysis in all swine.

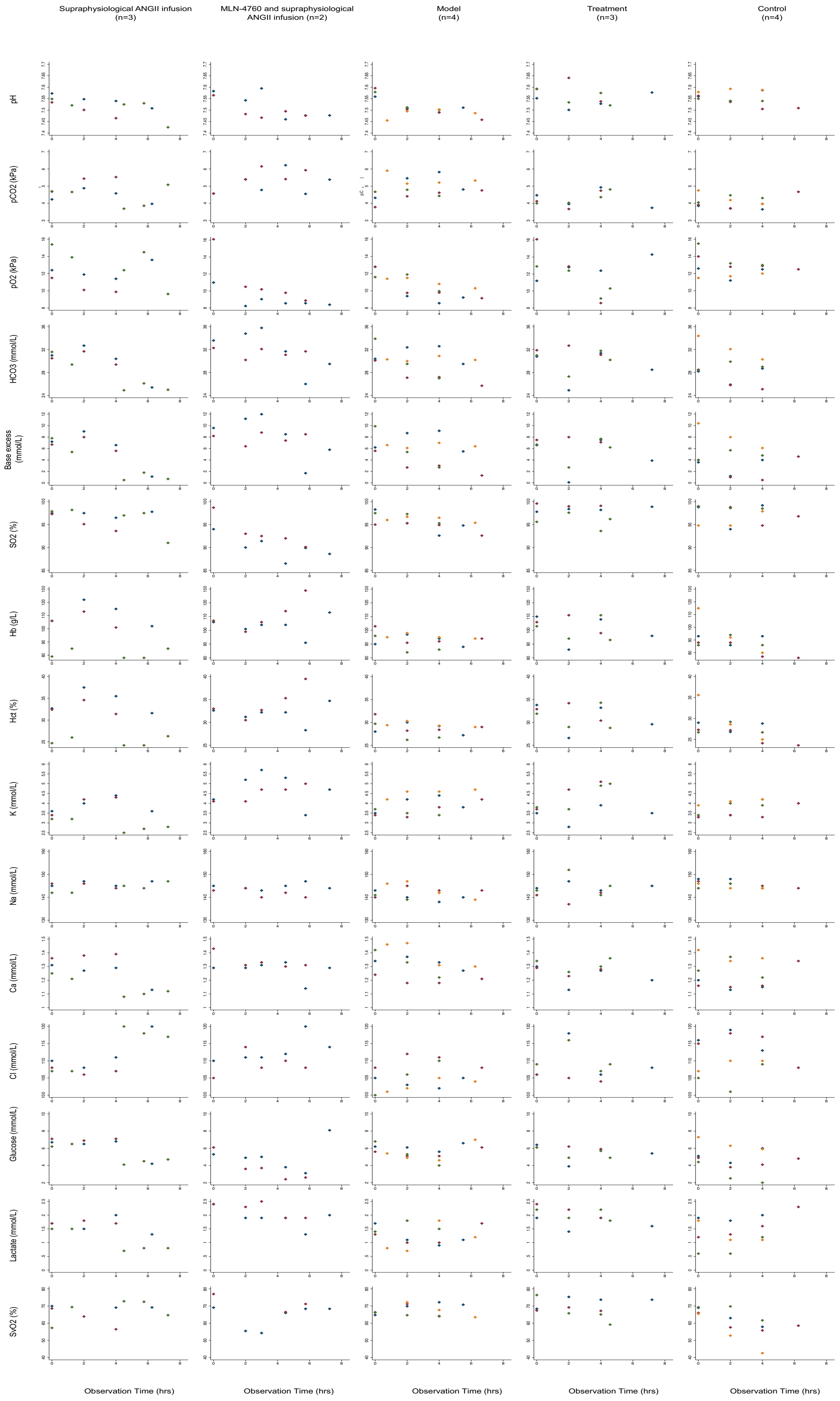

Supplementary Figure 2. The first column represents the swine infused with supraphysiological ANGII, the second column is MLN-4760 and supraphysiological ANGII infusion, the third column is MLN-4760 and low dose ANGII, the fourth column is MLN-4760, low dose ANGII and treatment with losartan and low molecular weight heparin, and the fifth column is control individuals. Each individual is color coded with the same color in the groups. Rows from the top are measurements from arterial blood; pH, $PaCO_2$, PaO2, HCO_3, base excess, SaO_2, Hb, Hematocrit, potassium, sodium, calcium, chloride, glucose, lactate, and finally SvO_2 measured from samples drawn from the Swan-Ganz catheter. Source data are provided as a source data file.

	Supraphysio-logical ANGII infusion (n=3)			MLN-4760 and supraphysio-logical ANGII infusion (n=2)		MLN-4760 and low dose ANGII infusion (n=4)				MLN-4760 low dose ANGII infusion and treatment (n=3)			Untreated sedated (n=4)			
**Swine**	**1**	**2**	**3**	**4**	**5**	**6**	**7**	**8**	**15**	**9**	**10**	**11**	**12**	**13**	**14**	**16**
P-CRP (mg/L)																
**Baseline**	<1	<1	<1													
**1**	<1	<1	<1													
**2**	<1	<1	<1													
**3**	<1		<1													
**4**			<1													
B-ESR (mm)																
**Baseline**				1	2	2	5	3		1	3	3	2	3		
**1**				3	3	1	3	3		1	3	3	2	3		
**2**				2	3	2	3	3		1	3	3	2	2		
**3**				3	2	2										
P-Albumin (g/L)																
**Baseline**	12	13	11	11	11	<10	11	12	11,40	13	13	12	11	12	10,70	12,70

1	10	13	12	<10	11	<10	10	10	11,00	11	11	12	10	11	9,30	12,20
2	<10	10	11	11	12	<10	<10	11	9,90	11	<10	<10	<10	10	8,20	
3	<10		10	11	<10	<10			10,00							
4			11						8,20							
P-Creatinine (µmol/L)																
**Baseline**	109	94	123	111	95	94	103	102	92,00	130	125	98	126	103	96,00	108,00
1	123	101	138	114	99	102	109	103	101,00	125	142	111	129	114	91,00	111,00
2	122	87	132	135	103	103	116	105	104,00	143	162	126	131	105	93,00	
3	130		130	134	122	106			119,00							
4			121						126,00							
P-AST (µkat/L)																
**Baseline**	0.45	0.42	0.5	0.38	0.43	0.41	0.41	0.4	0,33	0.45	0.37	0.4	0.46	0,33	0,42	0,42
1	0.47	0.43	0.6	0.38	0.49	0.48	0.48	0.46	0,62	0.4	0.3	0.58	0.45	0,36	0,65	0,46
2	0.56	0.42	0.54	0.68	0.58	0.61	0.55	0.46	0,53	0.64	0.39	0.63	0.43	0,44	0,59	
3	0.7		0.44	0.65	0.6	0.54			0,50							
4			0.48						0,44							
P-ALT (µkat/L)																
**Baseline**	1.77	1.47	1.52	0.94	1.2	1.34	1.4	1.07	1,27	1.34	1.05	0.89	1.1	1,18	1,12	0,93
1	1.72	1.39	1.67	0.79	1.14	1.34	1.33	0.95	1,38	1.16	0.86	0.9	1.01	1,03	0,98	0,94

2	1.46	1.19	1.53	1.02	1.28	1.38	1.2	1.01	1,26	1.18	0.88	0.93	0.89	1,03	1,00	
3	1.64		1.47	1.14	1.23	1.34			1,26							
4			1.44						1,15							
P-GGT (µkat/L)																
**Baseline**	0.4	0.51	0.56	0.78	1	0.75	0.67	0.88	0,54	1.2	1	0.56	1.6	0,66	0,63	0,59
1	0.39	0.5	0.63	0.84	0.78	0.73	0.76	0.96	0,55	1	0.78	0.64	1	0,58	0,56	0,61
2	0.35	0.42	0.6	0.87	0.94	0.96	0.88	0.84	0,54	1.3	0.92	0.62	0.77	0,59	0,55	
3	0.35		0.58	0.84	0.72	0.72			0,51							
4			0.59						0,54							
P-LD (µkat/L)																
**Baseline**	9.8	9.4	9.7	8.7	10.6	10.2	9.3	11.1	9,54	10.9	11	8.8	11.3	11	9,40	8,65
1	9.7	9.4	10.5	7.4	10.3	7.9	8.7	10	9,60	9.5	9.7	8.8	10.2	9,6	8,51	8,34
2	8.2	7.6	9.4	9.3	11.4	10.2	8.7	10.1	8,72	8.8	8.8	8.2	8.9	9,5	7,64	
3	8.6		8.7	9.2	11.6	10.2			8,75							
4			8.8						8,29							
P-CK (µkat/L)																
**Baseline**	9.7	16.5	12.6	4.6	9.1	8.1	9.2	5.8	6,10	4.7	4.6	4.9	7.9	5,1	6,91	4,36
1	7.9	12.4	13.7	3.7	11.3	7.9	8.6	5.2	6,65	4.3	3.7	5.4	7	3,7	6,29	4,02
2	6.1	8.8	10.4	4.4	12.1	7.6	8	5	5,68	4.5	4.9	5	7.1	3,8	5,54	

**3**	**6.7**		**6.8**	**5.8**	**15.2**	**6.6**			**5,46**							
**4**			**6.1**						**4,75**							
P-TG (mmol/L)																
**Baseline**	**0.27**	**0.25**	**0.21**	**0.32**	**0.22**	**0.18**	**0.17**	**0.31**	**0,15**	**0.24**	**0.19**	**0.13**	**0.21**	**0,25**	**0,28**	**0,25**
**1**	**0.46**	**0.56**	**0.26**	**0.35**	**0.3**	**0.22**	**0.37**	**0.36**	**0,38**	**0.21**	**0.16**	**0.2**	**0.3**	**0,39**	**0,55**	**0,26**
**2**	**0.51**	**0.38**	**0.32**	**0.43**	**0.43**	**0.19**	**0.24**	**0.47**	**0,45**	**0.3**	**0.17**	**0.22**	**0.26**	**0,59**	**0,62**	
**3**	**0.42**		**0.27**	**0.48**	**0.75**	**0.2**			**0,31**							
**4**			**0.36**						**0,31**							
P-Factor VIII (kIU/L)																
**Baseline**	**3.47**	**>1.8**	**>1.8**	**>1.8**	**>1.8**	**>1.8**	**>1.8**	**>1.8**		**>1.8**						
**1**	**3.78**	**>1.8**	**>1.8**	**>1.8**	**>1.8**	**>1.8**	**>1.8**	**>1.8**		**>1.8**						
**2**	**3.69**	**>1.8**	**>1.8**	**>1.8**	**>1.8**	**>1.8**	**>1.8**	**>1.8**								
**3**	**3.62**		**>1.8**			**>1.8**				**>1.8**						
P-VWF GP1bA (kIU/L)																
**Baseline**	**1.29**	**1.45**	**1.16**	**1**	**1.46**	**1.47**	**1.22**			**>2**	**>2**	**>2**				
**1**	**1.5**	**2.7**	**1.47**	**1.14**	**1.43**	**1.44**	**1.92**	**1.31**		**1.48**		**>2**				
**2**	**1.43**	**2.94**	**2.46**	**1.29**	**1.89**	**1.28**	**1.85**	**1.35**		**>2**						
**3**	**1.48**		**2.85**	**1.29**		**1.48**	**1.4**									

B-Platelet Count (x10^9/L)																
**Baseline**				427	381	250	368	382	370,0	401	385	492	319	367	416	276,0
**1**				294	312	246	369	307	379,0	339	298	429	255	234	338	265,0
**2**				325	425	257	363	301	354,0	263	248	401	191		318	
**3**				341	439	250			268,0							
**4**									309,0							
B-Reticulocytes (x10^9/L)																
Baseline						22	38	48	21,0	37	22	34		48	32	41,0
1						23	39	38	26,0	40	18	34		34	29	34,0
2						23	42	35	25,0	39	15	32	18		21	
3						24			24,0							
4									16,0							
B-IPF (%)																
**Baseline**						1.3	3.1	1		0.8	1	0.7	1.3	0,8		
**1**						1.4	2.3	1.2		1.2	1.6	0.8	1.5	1,3		
**2**						1.5	2	1.4		1.4	1.7	0.9	1.8			
**3**						1.4										

P-D-dimer (mg/L FEU)																
Baseline	**0.47**	**0.69**	**0.38**	**0.26**	**0.26**	**0.24**	**0.63**	**0.35**	**0,47**	**0.44**	**0.45**	**0.73**	**0.33**	**0,47**	**0,32**	**0,68**
1	**1.04**	**1**	**0.77**	**0.5**	**0.45**	**0.39**	**0.85**	**0.46**	**0,45**	**0.48**	**0.48**	**0.75**	**0.59**	**0,7**	**0,32**	**N/A**
2	**0.92**	**1.04**	**0.8**	**0.55**	**0.63**	**0.41**	**0.82**	**0.55**	**0,50**	**0.67**	**0.48**	**0.82**	**0.42**	**0,57**	**0,43**	**N/A**
3	**1.12**		**0.92**			**0.38**			**0,44**							
4									**0,40**							
P-Soluble fibrin (mg/L)																
Baseline		**<5**	**7**	**5**		**6**	**<5**	**<5**	**3,16**	**6**	**<5**	**<5**	**<5**	**<5**	**3,85**	**6,80**
1		**<5**	**14**	**7**	**17**	**8**	**5**	**8**	**3,63**	**<5**	**<5**	**<5**	**7**	**<5**	**5,95**	**N/A**
2		**<5**	**7**	**10**	**11**	**9**	**6**	**9**	**2,68**	**11**	**<5**	**<5**	**8**	**6**	**5,99**	
3			**9**			**19**			**10,09**							
4									**1,00**							
P-Fibrinogen (g/L)																
Baseline	**1.6**	**0.9**	**1.4**	**0.9**	**0.8**	**0.9**	**1**	**1.1**	**1,01**	**1.2**	**1.2**	**1.2**	**0.9**	**1**	**1,08**	**15,90**
1	**1.6**	**0.9**	**1.4**	**0.9**	**1.4**	**0.9**	**1.1**	**1**	**1,05**	**1.1**	**1**	**1.2**	**0.9**	**0,9**	**0,90**	**N/A**
2	**1.5**	**0.9**	**1.3**	**1.2**	**1.7**	**0.9**	**1**	**1.1**	**0,99**	**1.1**	**1.2**	**1.3**	**0.9**	**1**	**0,89**	
3	**1.8**		**1.1**			**0.9**			**1,05**							
4									**0,99**							

Deep venous thrombosis		**0**	**0**	**1**	**0**	**0**	**0**	**0**	**0**	**1**	**0**	**0**	**0**	**0**	**0**	**0**
Total Urinary output (ml/hour)	**327**	**112**	**353**	**178**	**3**	**73**	**45**	**13**	**33**	**21**	**28**	**153**	**387**	**130**	**120**	**208**
Bleeding Time (s)																
**Baseline**		**255**	**285**	**225**	**325**	**195**	**210**	**60**	**210**	**105**	**120**	**105**	**165**	**150**	**225**	**315**
**Mid**		**90**	**120**	**105**	**105**	**45**	**45**	**60**	**90**	**240**	**120**	**60**	**270**	**150**	**300**	**360**
**End**		**15**	**105**	**90**	**15**	**145**	**30**	**45**	**255**	**120**	**105**	**100**	**180**	**180**	**330**	**270**

Supplementary Table 1. ANGII: Angiotensin II; CRP: C-reactive Protein; ESR: Erythrocyte Sedimentation Rate; AST: Aspartate Aminotransferase; ALT: Alanine Aminotransferase; GGT: Gamma-Glutamyl Transferase; LD: Lactate Dehydrogenase; CK: Creatinine Kinase; TG: Triglyceride; VWF: Von Willebrand Factor; IPF: Immature Platelet Fraction. N/A: Not available due to coagulation in the test tube.

Outcome: PA systolic	Coef.	S.e.
Intercept	21.59	1.53
Group indicators (ref. category: Control group)		
Model group (1 if model, 0 otherwise)	7.14	2.17
Treatment group (1 if treatment, 0 otherwise)	4.94	2.33
Time (minutes, centered at three hours)	0.017	0.0029
Interaction group indicator and time<br>(ref. category: Control group)		
Model group (1 if model, 0 otherwise)	-0.0047	0.0045
Treatment group (1 if treatment, 0 otherwise)	-0.019	0.0044
Variance intercept	9.24	4.66
Variance time	0.000026	0.000018
Covariance intercept and time	-0.0025	0.0062
Log restricted-likelihood	-3735	
Number of animals	11	
Number of observations	1587	
Outcome: PaO_2	Coef.	S.e.
Intercept	12.72	0.44
Group indicators (ref. category: Control group)		
Model group (1 if model, 0 otherwise)	-2.13	0.60
Treatment group (1 if treatment, 0 otherwise)	-0.63	0.65

Variance intercept	1.05e-21	1.40e-20
Log restricted-likelihood	-71	
Number of animals	11	
Number of observations	39	
Outcome: O_2 saturation	Coef.	S.e.
Intercept	97.31	0.66
Group indicators (ref. category: Control group)		
Model group (1 if model, 0 otherwise)	-1.70	0.92
Treatment group (1 if treatment, 0 otherwise)	0.41	0.99
Variance intercept	0.96	0.86
Log restricted-likelihood	-75	
Number of animals	11	
Number of observations	39	
Outcome: $PaCO_2$	Coef.	S.e.
Intercept	4.09	0.17
Group indicators (ref. category: Control group)		
Model group (1 if model, 0 otherwise)	0.80	0.23
Treatment group (1 if treatment, 0 otherwise)	0.16	0.25
Variance intercept	0.057	0.055
Log restricted-likelihood	-27	

Number of animals	11	
Number of observations	39	
Outcome: Bleeding time	Coef.	S.e.
Intercept	239.72	35.22
Group indicators (ref. category: Control group)		
Model group (1 if model, 0 otherwise)	-156.58	50.30
Treatment group (1 if treatment, 0 otherwise)	-117.01	52.82
Time (minutes, centered at four hours)	-0.0031	0.11
Interaction group indicator and time (ref. category: Control group)		
Model group (1 if model, 0 otherwise)	-0.19	0.16
Treatment group (1 if treatment, 0 otherwise)	-0.058	0.16
Variance intercept	3940.37	2246.931
Variance time	2.99e-21	4.73e-20
Log restricted-likelihood	-298	
Number of animals	11	
Number of observations	56	

Supplementary Table 2. Mixed error-component model coefficients for PA systolic, O_2 saturation, PaO_2, $PaCO_2$ and bleeding time.
