# Supplementary material for: COVID-19 pathophysiology may be driven by an imbalance in the renin-angiotensin-aldosterone system
Source: Nat Commun. 2021 Apr 23;12:2417. doi: 10.1038/s41467-021-22713-z (PMC8065208; doi:10.1038/s41467-021-22713-z)
Supplement: Supplementary file 3 — Reporting Summary [file 41467_2021_22713_MOESM3_ESM.pdf]

nature research

Corresponding author(s): Johan Lundberg

Last updated by author(s): Feb 2, 2021

Reporting Summary

Nature Research wishes to improve the reproducibility of the work that we publish. This form provides structure for consistency and transparency in reporting. For further information on Nature Research policies, see our Editorial Policies and the Editorial Policy Checklist.

Statistics

For all statistical analyses, confirm that the following items are present in the figure legend, table legend, main text, or Methods section.

n/a	Confirmed	
☐	☒	The exact sample size (*n*) for each experimental group/condition, given as a discrete number and unit of measurement
☐	☒	A statement on whether measurements were taken from distinct samples or whether the same sample was measured repeatedly
☐	☒	The statistical test(s) used AND whether they are one- or two-sided<br>*Only common tests should be described solely by name; describe more complex techniques in the Methods section.*
☐	☒	A description of all covariates tested
☐	☒	A description of any assumptions or corrections, such as tests of normality and adjustment for multiple comparisons
☐	☒	A full description of the statistical parameters including central tendency (e.g. means) or other basic estimates (e.g. regression coefficient) AND variation (e.g. standard deviation) or associated estimates of uncertainty (e.g. confidence intervals)
☐	☒	For null hypothesis testing, the test statistic (e.g. *F*, *t*, *r*) with confidence intervals, effect sizes, degrees of freedom and *P* value noted<br>*Give P values as exact values whenever suitable.*
☒	☐	For Bayesian analysis, information on the choice of priors and Markov chain Monte Carlo settings
☒	☐	For hierarchical and complex designs, identification of the appropriate level for tests and full reporting of outcomes
☒	☐	Estimates of effect sizes (e.g. Cohen's *d*, Pearson's *r*), indicating how they were calculated

Our web collection on statistics for biologists contains articles on many of the points above.

Software and code

Policy information about availability of computer code

Data collection	Clinical MRI imaging was collected with the console software from the Philips Ingenia MRI scanner and the preclinical imaging used the console software from the Siemens MAGNETOM Aera 1.5 Tesla scanner.
Data analysis	Statistical analyses were performed using MATLAB (version R2020b, The Mathworks Inc., Natick, USA), IBM SPSS Statistics version 25 for Mac (IBM, Armonk, USA) and Stata 16.1 (StataCorp 2019, College Station, Texas, USA). A threshold of P<0.05 was considered statistically significant. Statistical analysis of manually drawn regions of interest in normal-appearing lung tissue on MRI perfusion were analyzed using MATLAB and imtool3D developed by Justin Solomon imtool3D (https://www.mathworks.com/matlabcentral/fileexchange/40753-imtool3d), MATLAB Central File Exchange, retrieved April 25, 2018) with further in-house development for 4D and ROI measurements.<br>The in house version can be accessed through github.com/SWICUrays/MRItools (https://doi.org/10.5281/zenodo.4492927). The code used for analysis has commit hash 63c46c4.<br>Extrapulmonary and extramediastinal tissues were manually masked. ROIs were manually selected in the superior portion of the pulmonary artery and in the aorta/innominate artery bifurcation. TTP and max enhancement parametric maps were generated by using the MATLAB function max and the color lookup table used for the figures is the parula color scheme from MATLAB. Mean relative contrast enhancement was defined as the enhancement between 10 to 25 seconds after the aortic peak divided by baseline signal during the initial 10 seconds. Stasis was estimated as the fraction with relative contrast enhancement above noise defined as three standard deviation of the signal during the measurement volumes. Other descriptive statistics on MRI perfusion were also generated in MATLAB. To generate fig. 7, curves were interpolated using the modified Akima method. This was done to generate a unified x-axis for simultaneous plotting. No interpolation was performed prior to calculating the functional ratio, mean TTP values or max relative enhancement of the lungs. Inter-rater agreement of CTPA measurements were calculated using the intraclass correlation coefficient of average measurements in SPSS.

For manuscripts utilizing custom algorithms or software that are central to the research but not yet described in published literature, software must be made available to editors and reviewers. We strongly encourage code deposition in a community repository (e.g. GitHub). See the Nature Research guidelines for submitting code & software for further information.

Data

Policy information about availability of data

All manuscripts must include a data availability statement. This statement should provide the following information, where applicable:

- Accession codes, unique identifiers, or web links for publicly available datasets
- A list of figures that have associated raw data
- A description of any restrictions on data availability

The data that support the findings of this study are available from the corresponding author upon reasonable request except for the patient imaging raw data due to privacy concerns.

Field-specific reporting

Please select the one below that is the best fit for your research. If you are not sure, read the appropriate sections before making your selection.

☒ Life sciences ☐ Behavioural & social sciences ☐ Ecological, evolutionary & environmental sciences

For a reference copy of the document with all sections, see nature.com/documents/nr-reporting-summary-flat.pdf

Life sciences study design

All studies must disclose on these points even when the disclosure is negative.

Sample size	We collected all RT-PCR-confirmed COVID-19 undergoing CTPA at Karolinska University Hospital in Huddinge, Stockholm, Sweden, between March 2nd (first patient admitted) and May 20th.
Data exclusions	CTPA scans were excluded due to motion artefacts (n = 47) and motion artifacts/angling problems (n=3) for the PA-diameter measurements. In the detection of embolism scans were excluded due to bolus timing issues (n=3). No animals were excluded. Exclusion criteria were not formally pre-established.
Replication	We did not have access to another CTPA cohort to try to replicate our findings. The biological replicates for the swine experimens were as follows; large animal infusion of supraphysiological levels of angiotensin II (n=3), large animal infusion of supraphysiological levels of angiotensin II with MLN-4760 ACE2 blocking (n=2), large animal low-rate infusion of angiotensin II with MLN-4760 ACE2 blocking (n=4), large animal low-rate infusion of angiotensin II with MLN-4760 ACE2 blocking plus treatment with losartan and LMWH (n=3) and, control animals sedated with sham infusions (n=4).<br>A minimum of four histological samples from lungs of all individuals were taken and sectioned. Samples were taken from the lower lobe at the dependent area, and roughly equidistant along the lateral wall to the lobe border and a final sample from the superior lobe. At least two samples were collected from the superior pole of the right kidney, inferior border of liver and small bowel with the most distension or discoloration that could be located. Representative macroscopic images including images of cut sections were acquired from three swine.
Randomization	For the clinical cohort we included every scan that was possible to measure. We provide descriptive information about the cohort and analys it as is. No co-variates are analysed. This part of the study was retrospective and, as such, can not be performed in randomization. The swines reported in this study are sequentially performed. Using information from the first experiments, we then planned the next set of experiments. As such, no randomization could be performed.
Blinding	For the aforementioned reasons of sequentially performing the experiments, no blinding was possible during the experiments.

Reporting for specific materials, systems and methods

We require information from authors about some types of materials, experimental systems and methods used in many studies. Here, indicate whether each material, system or method listed is relevant to your study. If you are not sure if a list item applies to your research, read the appropriate section before selecting a response.

Materials & experimental systems

n/a	Involved in the study	
☒	☐	Antibodies
☒	☐	Eukaryotic cell lines
☒	☐	Palaeontology and archaeology
☐	☒	Animals and other organisms
☐	☒	Human research participants
☒	☐	Clinical data
☒	☐	Dual use research of concern

Methods

n/a	Involved in the study	
☒	☐	ChIP-seq
☒	☐	Flow cytometry
☒	☐	MRI-based neuroimaging

Animals and other organisms

Policy information about studies involving animals; ARRIVE guidelines recommended for reporting animal research

Laboratory animals	Sixteen female swine with weights between 34 and 41 kg and age 12 +/- 2 weeks were used in this study.
Wild animals	No wild animals were used in this study.
Field-collected samples	No field-collected samples were used in this study.
Ethics oversight	All animal studies were conducted according to Karolinska Institutet guidelines for animal experiments. The study was approved by the Regional Ethics Committee for Animal Research in Stockholm, Sweden (no. 6716-2020).

Note that full information on the approval of the study protocol must also be provided in the manuscript.

Human research participants

Policy information about studies involving human research participants

Population characteristics	A consecutive cohort of all patients (83 females, 28%; mean age 59 years, standard deviation 16 years) with RT-PCR-confirmed COVID-19 undergoing CTPA at Karolinska University Hospital in Huddinge, Stockholm, Sweden.
Recruitment	Data collection took place between March 2nd (first patient admitted) and May 20th were retrospectively evaluated.
Ethics oversight	The human studies and case reports were approved by the Swedish Ethical Review Authority (no. 2020-01895 and no. 2020-01752); Informed consent was officially waived by the Review Authority due to the retrospective nature of the study, though written informed consent by next of kin was obtained for publication of the one clinical case of MRI lung perfusion and the other for the PA pressure measurements. The human studies and case reports were performed in compliance with the Helsinki declaration.

Note that full information on the approval of the study protocol must also be provided in the manuscript.
